# Safety-netting advice documentation in out-of-hours primary care: a retrospective cohort from 2013 to 2020

**DOI:** 10.3399/BJGP.2024.0057

**Published:** 2024-12-24

**Authors:** Peter J Edwards, Samuel Finnikin, Fay Wilson, Ian Bennett-Britton, Andrew Carson-Stevens, Rebecca K Barnes, Rupert A Payne

**Affiliations:** Centre for Academic Primary Care, Bristol Medical School, University of Bristol, Bristol and honorary research associate, Institute of Applied Health Research, University of Birmingham, Birmingham.; Institute of Applied Health Research, University of Birmingham, Birmingham.; Badger Group, Birmingham, UK.; Centre for Academic Primary Care, Bristol Medical School, University of Bristol, Bristol.; Primary and Emergency Care Research (PRIME) Centre, Division of Population Medicine, School of Medicine, Cardiff University, Cardiff.; Nuffield Department of Primary Care Health Sciences, University of Oxford, Oxford.; Exeter Collaboration for Academic Primary Care, University of Exeter Medical School, Exeter.

**Keywords:** after-hours care, documentation, health communication, medical records, patient education as topic, patient safety, problem-oriented, safety-netting, self care

## Abstract

**Background:**

Providing safety-netting advice (SNA) in out-of-hours (OOH) primary care is a recognised standard of safe care, but it is not known how frequently this occurs in practice.

**Aim:**

Assess the frequency and type of SNA documented in OOH primary care and explore factors associated with its presence.

**Design and setting:**

This was a retrospective cohort study using the Birmingham Out-of-hours general practice Research Database.

**Method:**

A stratified sample of 30 adult consultation records per month from July 2013 to February 2020 were assessed using a safety-netting coding tool. Associations were tested using linear and logistic regression.

**Results:**

The overall frequency of SNA per consultation was 78.0% (1472/1886), increasing from 75.7% (224/296) in 2014 to 81.5% (220/270) in 2019. The proportion of specific SNA and the average number of symptoms patients were told to look out for increased with time. The most common symptom to look out for was if the patients’ condition worsened followed by if their symptoms persisted, but only one in five consultations included a timeframe to reconsult for persistent symptoms. SNA was more frequently documented in face-to-face treatment-centre encounters compared with telephone consultations (odds ratio [OR] 1.77, 95% confidence interval [CI] = 1.09 to 2.85, *P* = 0.02), for possible infections (OR 1.53, 95% CI = 1.13 to 2.07, *P* = 0.006), and less frequently for mental (versus physical) health consultations (OR 0.33, 95% CI = 0.17 to 0.66, *P* = 0.002) and where follow-up was planned (OR 0.34, 95% CI = 0.25 to 0.46, *P*<0.001).

**Conclusion:**

The frequency of SNA documented in OOH primary care was higher than previously reported during in-hours care. Over time, the frequency of SNA and proportion that contained specific advice increased, however, this study highlights potential consultations where SNA could be improved, such as mental health and telephone consultations.

## Introduction

In England, out-of-hours (OOH) primary care is provided from 18:30 h to 08:00 h on weekdays, and all day on weekends and public holidays.^1^ It is typically commissioned from a specific OOH provider, who cover large geographical areas across multiple towns and cities, rather than the patient’s usual GP.^1^ Records from OOH encounters are typically sent to a patients’ usual GP who takes over responsibility for ongoing care once OOH time ends. A systematic review of primary care OOH in the UK and similar international settings reported that OOH usage is primarily driven by problems perceived as urgent by patients.^2^ Usage is more common by children <5 years of age, adults >65 years, women, those with higher socioeconomic deprivation, and those with chronic diseases or mental health problems.^2^ As a result of the scarcity of resources available, urgency of patients’ presentations, and unfamiliarity with patients, OOH care is perceived as being higher risk than routine practice.^3^

Safety-netting is recommended to help manage risk in multiple different UK guidelines,^4^^–^7 and is a key component of safe OOH care.^8^^,^^9^ The term most frequently refers to communication within a healthcare encounter, advising when and how patients should seek further medical help if their condition worsens, fails to improve, or they have new concerns about their health.^10^ However, in recent years the term ‘safety-netting’ has also been used to describe other activities outside of healthcare encounters.^11^^,^^12^ To avoid ambiguity, ‘safety-netting advice’ (SNA) is now the preferred term for the aforementioned communication within healthcare encounters.^4^ In addition to explaining when patients should reconsult, comprehensive SNA also includes explanations of what to expect, such as expected duration of symptoms, thus promoting appropriate health-seeking behaviours.^13^ Overarchingly, SNA is used to help mitigate the risk of serious harm from diagnostic uncertainty and when time is used as a diagnostic tool.^13^ Consequently, the provision and documentation of SNA is an auditable standard in OOH primary care.^9^

Research into primary care safety-netting practices has predominantly assessed in-hours care, although a minority has been in OOH.^12^^,^^14^ Qualitative interview studies have highlighted the importance of SNA in the OOH setting,^15^^,^^16^ and a belief that the quality of SNA during OOH may be more comprehensive than during in-hours practice.^17^ This may be because GPs more frequently provide SNA in single-problem consultations^18^ and for acute problems,^19^ which are more common in the OOH setting.^2^ However, poor documentation of SNA has been reported in a qualitative interview study involving OOH clinicians,^17^ and a quantitative in-hours study,^18^ but there have been no large quantitative reports on how SNA is documented in OOH. Fully documenting verbal SNA is important to ensure safe handover of patients to their usual GP, and for medicolegal purposes as GPs have been reprimanded for not documenting their spoken SNA.^20^ OOH SNA may also differ from in-hours practice, as the role of OOH providers is to deliver immediate short-term medical care, until the patient’s usual GP re-opens.

**Table:** How this fits in

Previous research has reported on safety-netting advice (SNA) documented in patient records during in-hours practice but this, to the authors’ knowledge, is the first large-scale (>1000 consultations) longitudinal analysis of the type of safety-netting documented advice during out-of-hours (OOH) primary care. This study demonstrated an increasing frequency of documented SNA in OOH records and increasing utility of specific advice over time. In contrast to previous reports of verbalised safety-netting during in-hours practice, this study found a higher frequency of SNA in records from face-to-face compared with telephone encounters. This study also showed safety-netting advice was more likely to be documented for patients with possible infections, but less frequently for mental health consultations. That is a possible area for improvement, in line with current UK policy for ‘parity of esteem’ between physical and mental health conditions.

The aims of this retrospective cohort study was to describe how and when SNA was documented for adult patients being managed in OOH primary care, describe any changes in practice over time, and explore factors associated with the presence of SNA. This information could then be used to explore possible areas for improving care.

## Method

### Data

This study was a retrospective cohort review of OOH electronic health records (EHRs) using the Birmingham Out-of-hours general practice Research Database (BORD).^21^^,^^22^ BORD is a database comprising all clinical encounters from July 2013 to July 2020 (*n* = 694 198) from a single OOH primary care provider (Badger Group) in the West Midlands, England, serving a population of approximately 1.4 million people. Data in BORD were extracted from the Adastra EHR system^23^ and includes free-text entries as well as coded data.

### Stratified sample

**Figure 1.**
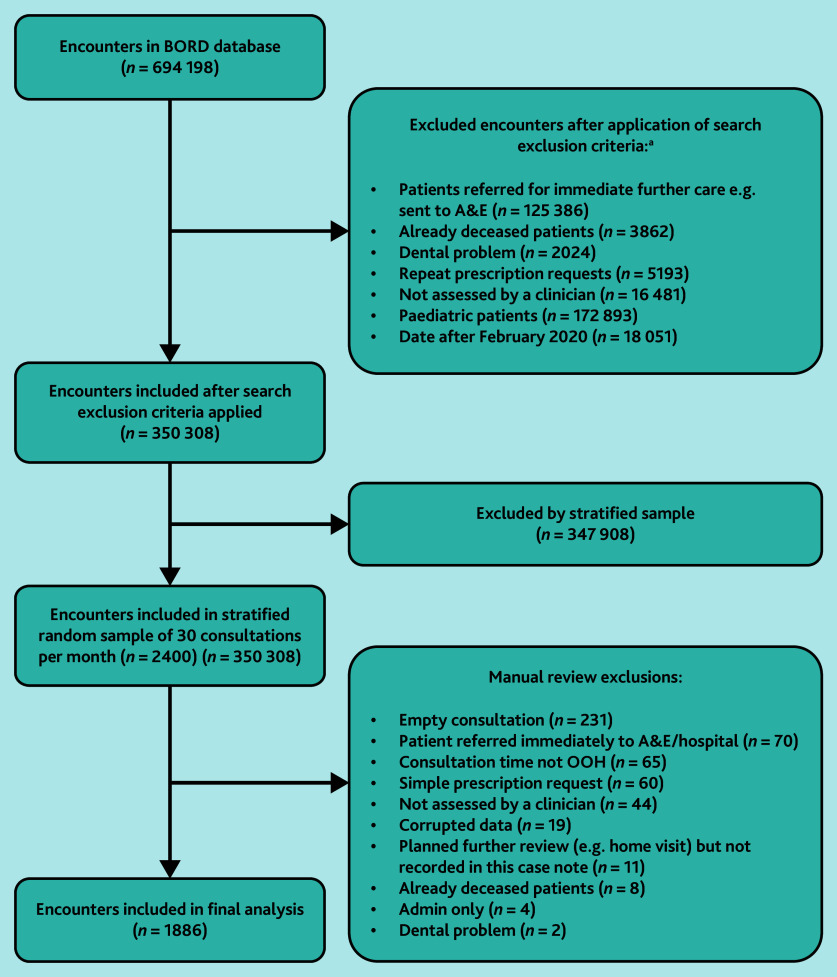
Consolidated Standards of Reporting Trials (CONSORT) diagram. aExclusions applied in descending order. A&E = accident and emergency. BORD = Birmingham Out-of-hours general practice Research Database. OOH = out-of-hours.

A stratified sample of 30 consultations per month (comprising 10 home visits, 10 telephone consultations and 10 treatment-centre encounters) was obtained from July 2013 to February 2020 (inclusive) after coded exclusion criteria were applied, and consultations were then manually reviewed for further exclusion criteria (Figure 1).

### Coding

A Safety-Netting Coding Tool^10^^,^^24^ (SaNCoT) was optimised for large-scale coding of the free-text of OOH records (version 2.0 see Supplementary Information S1 and Supplementary Figure S1a & S1b). All consultations that contained SNA underwent detailed coding (see Supplementary Information S1 and Supplementary Figure S1b and codebook). Problems were classified using the International Classification of Primary Care Third Edition (ICPC-3).^25^^,^^26^

The full inclusion and exclusion criteria for classifying SNA are reported in the screening instructions (see Supplementary Information S2). Of note, SNA was distinguished from follow-up, in that a symptom or condition had to occur (‘worsening’, ‘not better in 2 weeks’) to prompt a medical review, whereas follow-up happens regardless of any change to the patient’s symptoms. Follow-up was coded as present if either known (‘has GP appointment next week’) or recommended (‘advised to review with own GP’) follow-up was documented. Coders could also select the type of follow-up recommended (see Supplementary Figure S1a).

A pilot study of 100 random consultations, stratified by consultation type, was conducted to test the interrater reliability (IRR) of the updated SaNCoT. In total, 87/100 consultations met the inclusion criteria after manual review. Two coders (the first and second authors) independently screened consultations for the presence of SNA and follow-up with agreement scores of 98% (Cohen’s kappa [κ] = 0.93) and 91% (κ = 0.82), respectively. Overall agreement scores for full coding of 69 consultations that contained SNA was 89% (κ = 0.74, unweighted agreement) and 94% (κ = 0.75, weighted agreement, see Supplementary Tables S1 and S2).

### Software and statistical analysis

Data were exported into Stata/MP 17.0 for analysis and the executed code is available.^27^ Univariable and multivariable logistic regression models were used to test associations for binary outcomes (SNA present or absent, SNA contains specific advice or generic only, prescription issued or not) and generate odds ratios (OR) with 95% confidence intervals (CIs). In multivariable modelling, consultations with missing data were excluded and multilevel mixed effects were used to adjust for clusters of patients seen by the same clinician. Univariable linear regression was used to test associations for continuous outcomes (number of symptoms listed in SNA). Power calculations are available in Supplementary Information S3.

## Results

### Participant and consultation characteristics

Of the 2400 consultations sampled, 514 consultations were excluded after manual review (see Supplementary Table S3 for reasons). Table 1 describes the included participant and consultation characteristics. There were 1886 consultations with 1862 unique patients. There were more female patients (62.9%, 1171/1862) and disproportionately more patients from very deprived backgrounds (Townsend quintile 5, 37.3%, 695/1862) . The mean patient age was 57.8 years (standard deviation [SD] 24.7, range 16–102); this was higher for patients assessed on home visits (mean 78.2 years, SD 14.4), rather than by telephone (mean 54.4 years, SD 24.3) or in treatment centres (mean 39.8 years, SD 6.8).

**Table 1.** Consultation and patient characteristics (*n* = 1886 consultations with 1862 unique patients and 353 clinicians)^a^

**Patient or consultation characteristic**	***N***	**%**
**Patient sex**		
Male	690	37.1
Female	1171	62.9
Not reported	1	0.1

**Patient age**		
16–34	460	24.7
35–64	567	30.5
≥65	835	44.8

**Patient Townsend quintile**		
1st (least deprived)	219	11.8
2nd	257	13.8
3rd	285	15.3
4th	354	19.0
5th (most deprived)	695	37.3
Not reported	52	2.8

**Consultation type**		
Telephone	480	25.5
Home visit	701	37.2
Treatment centre	705	37.4

**Problems per consultation**		
1	1732	91.8
2	141	7.5
3	11	0.6
4	2	0.1

**Consultations per clinician**		
1	148	41.9
2	57	16.1
3–4	52	14.7
5–9	39	11.0
10–19	33	9.3
20–44	24	6.8

a *Twenty patients had two consultations and two patients had three consultations. Five consultations had missing clinician identifiers.*

Consultations were undertaken by 353 unique clinicians, with a median of two consultations per clinician (range 1–44) and most consultations (91.8%, 1732/1886) were for one problem only.

### SNA frequencies

The overall frequency of SNA was 78.0% (1472/1886) per consultation and 77.3% (1588/2055) per problem. Supplementary Table S4 describes the SNA and follow-up frequencies for problem types. SNA was more frequently used over follow-up (77.3%, 1588/2055 versus 43.9%, 902/2055, respectively per problem). For problem categories with *n*>50, respiratory had the highest frequency of SNA (85.6%, 332/388) and psychological had the lowest (51.9%, 40/77).

### Content of SNA

Table 2 describes the content of documented SNA. Per consultation, most SNA applied to the problem only (51.1%, 752/1472) and followed a ‘conditional plus course of action’ format (96.1%, 1415/1472) [‘if x happens do y’]. It was slightly more common for SNA to contain generic advice only (52.7%, 776/1472) [‘call back if worsens’], rather than some specific advice (47.3%, 696/1472) [‘if no better in 2 days then see GP’]. Of 1472 consultations, most patients were advised to seek help in one (59.6%, *n* = 877) or two (32.7%, *n* = 482) locations, although 56 consultations specified three to four locations (3.8%, *n* = 1472). The most common location was to recontact the OOH team (64.8%, 955/1472), followed by the patient’s own GP (35.9%, 529/1472), unspecified ‘medical help’ or similar (22.3%, 328/1472), and emergency services (12.1%, 178/1472). It was rare (0.2%, 3/1472) that clinicians recorded providing written SNA.

**Table 2.** Content of documented safety-netting advice (SNA)^a,b^

**SNA coding question and codes from reviewing** **medical records**	**Consultations (*n* = 1472)**		**Problems (*n* = 1588)**	

	***N***	**%**	***N***	**%**
**Problem or treatment SNA**				
Problem only: *‘if it gets worse’*	752	51.1	792	49.9
Treatment/management plan only: *‘if the tablets cause s/e’*	4	0.3	4	0.3
Both/mixture: *‘if it gets worse or tablets cause s/e’*, *‘any concerns’*	716	48.6	792	49.9

**Format**				
Symptom/conditional + action: *‘if x happens do y’*	1415	96.1	1528	96.2
Symptom/conditional warning only: *‘worsening advice given’*	57	3.9	60	3.8

**Number of different symptoms/conditions to look out for**				
1	534	36.3	586	36.9
2	397	27.0	431	27.1
3–4	313	21.3	342	21.5
5–6	141	9.6	142	8.9
7–8	56	3.8	57	3.6
9–10	25	1.7	24	1.5
11–12	6	0.4	6	0.4

**Generic or specific advice**				
Generic only: *‘problems, issues, concerns, worsening’*, *‘not better’* [without time course]	776	52.7	854	53.8
Specific only: *‘coughs up blood, chest pain’*, *‘not better in 2 weeks’*	190	12.9	197	12.4
Both generic and specific	506	34.4	537	33.8

**Where to seek medical help** (may include multiple different actions in single consultation)				
No specified action: *‘worsening advice given’*	57	3.9	60	3.8
Unspecified medical help: *‘r/v inb’*, *‘seek medical attention if x’*	328	22.3	343	21.6
Own GP/practice	529	35.9	559	35.2
Recontact OOH service/111	955	64.9	1044	65.7
Emergency services	178	12.1	191	12.0
Other	23	1.6	27	1.7

**How many different locations to seek medical help**				
0	57	3.9	60	3.8
1	877	59.6	955	60.1
2	482	32.7	512	32.2
3	52	3.5	57	3.6
4	4	0.3	4	0.3

**Timescale of action** (may include multiple different timescales in single consultation)				
Not specified	1171	79.6	1275	80.3
Set time period: *‘if not better in 2 weeks’*, *‘more unwell over the weekend’*	239	16.2	252	15.9
Immediate/urgent: *‘see stat if x’*	82	5.6	83	5.2

**Written SNA**				
No evidence of written advice	1469	99.8	1585	99.8
Evidence of written advice	3	0.2	3	0.2

a *Percentages derived from n = 1472 consultations with SNA which applied to 1588 problems.*

b *Quotes are examples. OOH = out-of-hours. r/v inb = review if not better. s/e = side effects. Stat = immediately (from latin STATim)*.

Overall, there were 3871 symptoms/conditionals listed to look out for across 1472 consultations (see Table 3 for details). The mean number of different symptoms patients were told to look out for was 2.63 (SD 1.99, range 1–12, median 2, mode 1) per consultation that included SNA. The most common type of symptom documented was a new specific symptom or condition (31.1%, 1202/3871), followed by ‘worsening of symptoms/condition’ (16.9% 653/3871) then ‘persistence of the current illness’ (16.7%, 647/3871). However, the most common type of symptom per consultation was if the patient’s condition worsened (43.4%, 639/1472). This was because it was common for clinicians to list multiple different new specific symptoms in a single SNA episode. In 622 of 1472 consultations patients were advised to seek help if their symptoms persisted, but in only 19.9% (124/622) of these was a timeframe recorded.

**Table 3.** Safety-netting advice (SNA) conditions/symptoms to look out for

**Condition/symptom category**	**Total symptoms** **(*n* = 3871)**		**Per consultation with** **SNA (*n* = 1472)**	

	***N***	**%**	***N***	**%**
**Specific symptom or condition**	1202	31.1	486	33.0
Other new symptom^a^	654	16.9	337	22.9
Fever(s)/temp(s)/rigor(s)/chill(s)^a^	170	4.4	150	10.2
Shortness of breath^a^	157	4.1	133	9.0
Pain including site specific pain^a^	125	3.2	119	8.1
Vomit^a^	86	2.2	86	5.8
Sepsis^a^	10	0.3	10	0.7

**Worsening (non-specified)**	653	16.9	639	43.4

**Current illness/symptoms** **persist**	647	16.7	622	42.3

**Concern/worries**	405	10.5	373	25.3

**Increase in pre-existing symptom**	207	5.3	188	12.8

**Red flags/‘when’ to seek help** **highlighted**	137	3.5	133	9.0

**Necessary/requires/wants/can/further advice/need(s)/etc**	132	3.4	127	8.6

**SOS/PRN**	123	3.2	123	8.4

**Unwell**	92	2.4	92	6.3

**New ‘symptom(s)’ or similar**	80	2.1	80	5.4

**‘Safety-netted’ or similar**	70	1.8	70	4.8

**Problem(s)/issue(s)**	57	1.5	57	3.9

**Change(s)**	40	1.0	40	2.7

**Return of pre-existing symptom**	26	0.7	26	1.8

a *Subcategory. SOS = as necessary (from latin si opus sit). PRN = as needed (from latin pro re nata). Temp = temperature.*

### Factors associated with SNA

Table 4 describes variables associated with the presence of SNA. Univariable modelling demonstrated associations with increased SNA and calendar year (OR 1.07 per year increase, 95% CI = 1.01 to 1.13, *P* = 0.025); treatment-centre consultations compared with telephone consultations (OR 2.58, 95% CI = 1.94 to 3.43, *P*<0.001), home visits compared with telephone consultations (OR 1.45, 95% CI = 1.11 to 1.88, *P* = 0.006), consultations where a prescription was issued (OR 1.96, 95% CI = 1.56 to 2.46, *P*<0.001), and for possible infections (OR 2.00, CI = 1.57 to 2.49, *P*<0.001). Associations were found between decreased SNA and the presence of documented follow-up (OR 0.44, 95% CI = 0.36 to 0.56, *P*<0.001); mental health compared with physical health consultations (OR 0.27, 95% CI = 0.16 to 0.45, *P*<0.001); and consultations for patients aged ≥65 years (OR 0.73, 95% CI = 0.59 to 0.91, *P* = 0.005).

In multivariable adjusted modelling, the associations remained for treatment-centre consultations compared with telephone consultations (OR 1.77, 95% CI = 1.09 to 2.85, *P* = 0.020), possible infections (OR 1.53, 95% CI = 1.13 to 2.07, *P* = 0.006), mental health consultations (OR 0.33, 95% CI = 0.17 to 0.66, *P* = 0.002), and the presence of follow-up (OR 0.34, 95% CI = 0.25 to 0.46, *P*<0.001). The association of increasing SNA over time appeared to persist although the association was weaker (OR 1.07 per year increase, 95% CI = 0.99 to 1.16, *P* = 0.094).

**Table 4.** Consultation and patient factors associated with safety-netting advice (SNA)^a^

**Covariate**	**Univariable modelling (*n* = 1886)**					**Multivariable modelling (*n* = 1827)**		

	***N***	**% SNA**	**OR**	**95% CI**	***P*>*value***	**OR**	**95% CI**	***P*>*value***
**External consultation factors**								
Year of consultation, per year increase in SNA	1886	78.0	1.07	(1.01 to 1.13)	0.025^b^	1.07	(0.99 to 1.16)	0.094^c^
Season of year								
Winter	508	77.6	1			1		
Spring	432	77.5	0.99	(0.73 to 1.34)	0.939	0.99	(0.67 to 1.46)	0.961
Summer	462	79.0	1.08	(0.79 to 1.46)	0.637	1.14	(0.77 to 1.67)	0.509
Autumn	484	77.9	1.01	(0.75 to 1.36)	0.959	1.15	(0.79 to 1.68)	0.468
Contact type								
Telephone	480	69.4	1			1		
Treatment centre	705	85.4	2.58	(1.94 to 3.43)	<0.001^b^	1.77	(1.09 to 2.85)	0.020^b^
Home visit	701	76.6	1.45	(1.11 to 1.88)	0.006^b^	1.32	(0.87 to 1.98)	0.188

**Internal consultation factors**								
Follow-up present								
No follow-up	1055	84.2	1			1		
Follow-up present	831	70.3	0.44	(0.36 to 0.56)	<0.001^b^	0.34	(0.25 to 0.46)	<0.001^b^
Prescription issued								
No prescription	1010	72.9	1			1		
Prescription issued	876	84.0	1.96	(1.56 to 2.46)	<0.001^b^	0.92	(0.64 to 1.34)	0.664
Infection								
Not suspected infection	1011	72.8	1			1		
Possible Infection	875	84.1	1.98	(1.57 to 2.49)	<0.001^b^	1.53	(1.13 to 2.07)	0.006^b^
Mental health								
Physical health problems	1808	79.2	1			1		
Mental health only	65	50.8	0.27	(0.16 to 0.45)	<0.001	0.33	(0.17 to 0.66)	0.002^b^
Mental and physical health	12	58.3	0.37	(0.12 to 1.16)	0.089	0.26	(0.06 to 1.09)	0.064^c^
Contains social only	1	–	–	–	–			

**Patient factors**								
Age category								
16–64 years	1040	80.5	1			1		
≥65 years	846	75.1	0.73	(0.59 to 0.91)	0.005^b^	0.86	(0.60 to 1.22)	0.393
Sex								
Female	1190	78.6	1			1		
Male	695	77.1	0.92	(0.73 to 1.15)	0.464	0.99	(0.75 to 1.32)	0.972
Townsend quintile								
1 (least deprived)	220	81.4	1			1		
2	259	79.2	0.87	(0.55 to 1.37)	0.545	0.75	(0.43 to 1.30)	0.298
3	290	74.1	0.66	(0.43 to 1.01)	0.055^c^	0.66	(0.39 to 1.12)	0.122
4	359	77.4	0.79	(0.52 to 1.20)	0.261	0.78	(0.46 to 1.31)	0.350
5 (most deprived)	706	77.6	0.79	(0.54 to 1.16)	0.239	0.70	(0.44 to 1.13)	0.149

a *Missing data: sex n = 1, Townsend n = 52, Wald test across Townsend quintiles for multivariable model P = 0.59.*

b *P<0.05*.

c *P<0.10.*

*CI = confidence interval. OR = odds ratio.*

As such a strong association between prescriptions and SNA was attenuated in the multivariable model, additional analysis to explore confounding relationships was undertaken (Supplementary Table S5). This demonstrated significantly fewer prescriptions for telephone consultations (7.5%, 36/480), compared with home visits (44.1%, 309/701 OR 9.04, 95% CI = 5.44 to 15.0, *P*<0.001) and treatment centres (75.3%, 531/705 OR 66.3 95% CI = 38.0 to 115.9, *P*<0.001).

When home visits and treatment-centre encounters were combined and considered together as a face-to-face encounter, this was associated with increased SNA in both univariable (*P*<0.001) and multivariable modelling (OR 1.46, 95% CI = 1.01 to 2.12, *P* = 0.044, Supplementary Table S6).

### Doctors compared with nurses

There was no significant difference between the frequency of SNA documented for patients seen by nurses (87.9%, 218/248) compared with doctors at treatment centres (84.4%, 362/429) (OR 1.64, 95% CI = 0.84 to 3.19, *P* = 0.15, Supplementary Table S7).

### Changes in SNA over time

**Figure 2.**
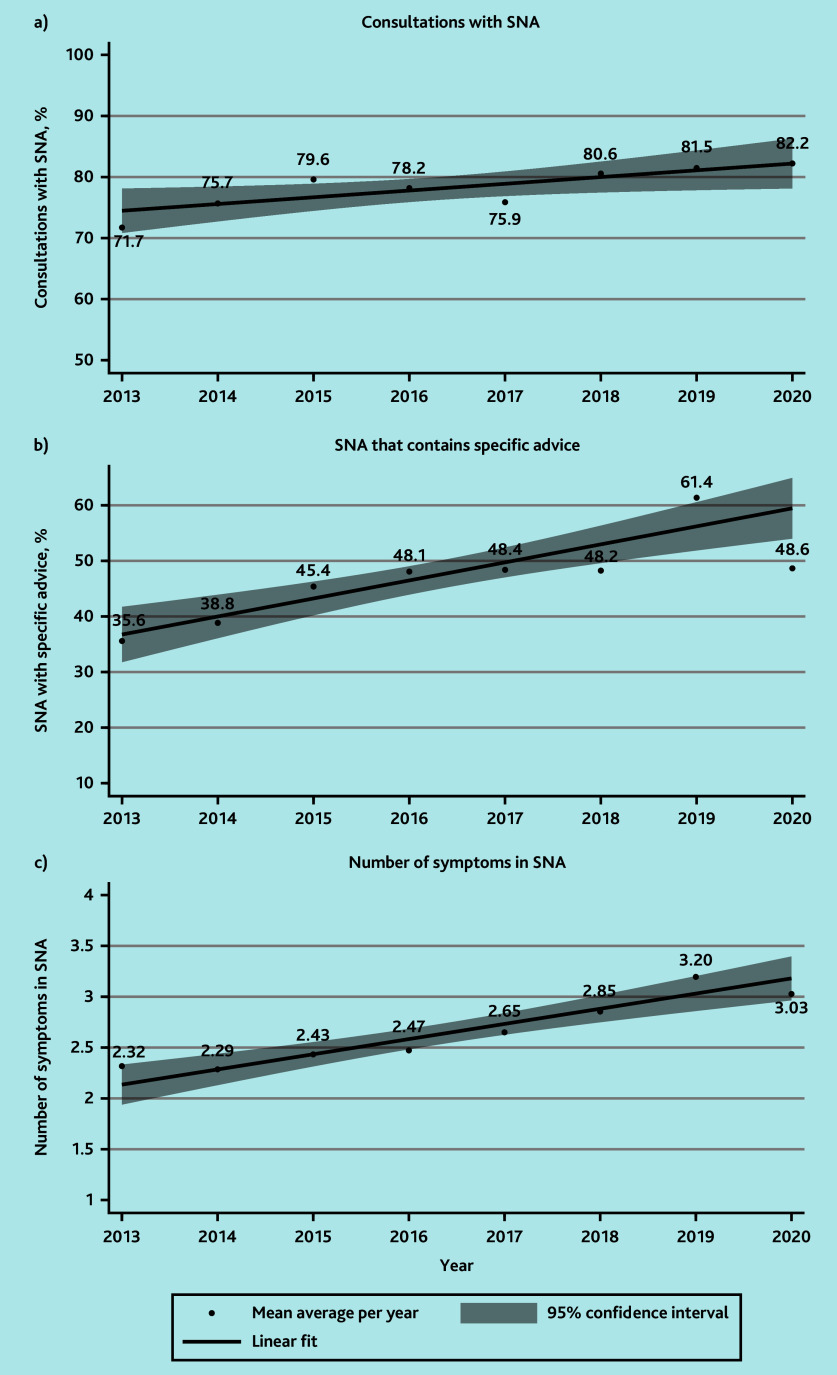
Safety-netting advice (SNA) over time. a) Consultations with SNA. b) SNA that contains specific advice. c) Number of symptoms in SNA. Data plotted from July 2013 to February 2020 (inclusive).

The frequency of SNA increased with time (Figure 2a) from 75.7% (224/296) in 2014 to 81.5% (220/270) in 2019 (range with full-year data available). The proportion of SNA that contained specific advice (univariable regression OR 1.14 per year increase, 95% CI = 1.08 to 1.20, P<0.001), and the average number of symptoms patients were told to look out for (β = 0.15, 95% CI = 0.10 to 0.20, P<0.001) also increased with time, as demonstrated in Figures 2b and 2c, respectively.

## Discussion

### Summary

In this retrospective cohort from 2013 to 2020, over time, there was an increase in the frequency of SNA documented in OOH records, the proportion of consultations containing specific SNA and the number of symptoms patients were advised to look out for. The most common symptom recorded in the SNA per consultation was if the patient’s condition worsened, followed by if their current illness persisted but only one in five consultations recorded a specific timeframe to reconsult for persistent symptoms. Most frequently, the records indicated patients were advised to re-seek medical help with the OOH provider followed by their own GP. Respiratory problems had the highest frequency of SNA, and consultations for mental health problems had less SNA than physical problems. SNA was more common in consultations for potential infections, and when patients were assessed face-to-face rather than by telephone.

### Strengths and limitations

To the authors’ knowledge, this is the first large-scale detailed analysis of documented SNA. This is also the first study, to the authors’ knowledge, to examine how SNA documentation has changed over time and explore factors associated with its presence in OOH. The free-text of every consultation was manually reviewed and the pilot study demonstrated ‘almost perfect’^28^ (κ>0.8) IRR in the screening process and ‘substantial’ agreement (κ = 0.61 to 0.80) for the application of the coding tool. Robust statistical methods were used to adjust for potential confounders and individual clinician practice and the distribution of patients’ age and deprivation quintile was consistent with a study of 242 373 consultations from BORD, suggesting the current sample was demographically representative of the larger dataset.^21^ However, the sampling of an equal number of consultations across modalities to maximise statistical power *—* instead of proportionally representing their occurrence in the database — necessitates cautious interpretation of the overall frequency of SNA as an estimate for the whole database. Nevertheless, the estimates for each consultation modality are likely to be accurate after applying the exclusion criteria.

One limitation is the study only evaluated documented SNA, and previous reports have highlighted SNA is often poorly documented.^17^^,^^19^ That said, studies exploring factors associated with the presence of SNA have found similar results when comparing documented to verbalised SNA.^18^

Although this study involved 353 clinicians, all were from the same OOH organisation and their practice may differ from clinicians at other OOH providers. This study included consultations up to March 2020 as during this month England entered the first national ‘lockdown’ in response to the COVID-19 pandemic, where protocols for healthcare encounters changed significantly.^29^ Despite the removal of COVID-19 restrictions in England, the use of remote consulting in-hours remains more prevalent than pre-pandemic.^30^ National data for OOH consultations are not published but it is possible this trend is mirrored in OOH care, potentially influencing the acuity of patients seen in different modalities, and having an impact on SNA practices.

### Comparison with existing literature

One previous study has reported on the frequency and type of SNA documented during in-hours GP consultations using data collected in 2014–15.^18^ Comparing results from the current study restricted to these years, the frequency of documented SNA in OOH consultations (77.6%, 462/595) was higher than in-hours (31.9%, 94/295) which is consistent with previous retrospective qualitative accounts.^17^ There was also a higher proportion of specific SNA documented during the current study in these years (41.5%, 207/499 problems), compared with in-hours (22.9%, 24/105 problems), and the mean number of symptoms to look out for was higher OOH (2.3) compared with in-hours (1.4).

A Flemish study^31^ of 77 OOH GP consultations for respiratory tract infections (RTIs) video-recorded in 2018, reported lower verbalised SNA (76.6%) and much lower free-text documentation SNA (14.5%) than SNA documented in the current study, which when restricted to just RTIs was 264/309 problems (85.4%).

The current study found lower frequencies of documented SNA in telephone consultations (69.4%, 333/480) compared with face-to-face encounters (81.0%, 1139/1406), which is the opposite of what Hammersley and colleagues^32^ found when assessing verbalised SNA in recordings of telephone (96.2%, 51/53) and face-to-face in-hours GP consultations (88.2%, 45/51) collected in 2017–18. One possible explanation is that the OOH provider audio-records telephone calls but not face-to-face encounters, thus clinicians may have been more thorough in their face-to-face documentation in case of an adverse advent.

### Implications for research and practice

Although this study has highlighted increased documentation of SNA in the OOH setting compared with in-hours there are still areas for potential improvement. First, instructing patients to seek help if their current symptoms persisted was the second most common form of SNA per consultation but in four of five cases no timeframe was recorded. Patients have previously voiced dissatisfaction around not knowing how long to wait to return with persistent symptoms and having a clear timeframe empowers self-care and helps patients accept responsibility for their follow-up plans.^33^^,^^34^ The authors of the current study recommend that SNA about persistent symptoms includes a timeframe and that this is documented.

Second, just over half of SNA remains generic only. There are still questions whether generic SNA — such as ‘any problems come back’ — is too vague to be useful,^35^ although some patients have reported it helps them feel they have ‘permission’ to seek help.^13^ However, a qualitative study on safety-netting for lung cancer symptoms reported that some patients experienced generic SNA as dismissive.^36^ The authors of the current study recommend clinicians aim to provide SNA with some specific elements in line with best practice,^13^ as generic advice alone may be perceived as the clinician terminating the consultation^37^ and may not be explanatory enough to enable patients to take responsibility for when they should reconsult.^33^

Third, as reported during in-hours care, written communication of SNA remains rare,^19^ despite patient demand for this format.^38^^–^40 Previous research has reported reasonable recall of SNA from face-to-face (68%) and telephone consultations (82%),^41^ but given approximately a fifth of consultations in this study (*n* = 364) documented four or more symptoms for patients to look out for, the authors of the current study recommend the increased utility of written advice to aid communication. Text messages with SNA have been shown to be acceptable to GPs^42^ and links to patient information leaflets or websites may have the added benefit of providing more specific SNA.

Fourth, the current finding of lower SNA in telephone encounters and consultations for mental health problems could be an area where additional educational resources are targeted. There may be unadjusted confounding factors behind these associations that further research could investigate, but clinicians should be aware that inadequate safety-netting has been implicated in safety incidents of remote consultations that resulted in serious harm and death.^43^

Finally, for optimal efficiency, auditing of records would be automated. This is more challenging for free-text rather than coded data, but there is increasing applications of artificial intelligence to analyse free-text data.^44^ This study provides a potential training dataset for a machine learning program that could use natural language processing techniques to achieve automation. This could then be used to provide real-time feedback to clinicians on their documentation of SNA and provide prompts to enhance patient safety, but it would need to be rigorously evaluated to ensure its safety, ethics, and legality.^45^

## Data

BORD is a controlled dataset. For enquires related to accessing BORD please contact Samual Finnikin: s.j.finnikin@bham.ac.uk.
